# Pathogenesis of *Neisseria gonorrhoeae* and the Host Defense in Ascending Infections of Human Fallopian Tube

**DOI:** 10.3389/fimmu.2018.02710

**Published:** 2018-11-21

**Authors:** Jonathan D. Lenz, Joseph P. Dillard

**Affiliations:** Department of Medical Microbiology and Immunology, University of Wisconsin-Madison, Madison, WI, United States

**Keywords:** *Neisseria gonorrhoeae*, fallopian tube, oviduct, organ culture, tissue explant, peptidoglycan, cilia, pelvic inflammatory disease

## Abstract

*Neisseria gonorrhoeae* is an obligate human pathogen that causes mucosal surface infections of male and female reproductive tracts, pharynx, rectum, and conjunctiva. Asymptomatic or unnoticed infections in the lower reproductive tract of women can lead to serious, long-term consequences if these infections ascend into the fallopian tube. The damage caused by gonococcal infection and the subsequent inflammatory response produce the condition known as pelvic inflammatory disease (PID). Infection can lead to tubal scarring, occlusion of the oviduct, and loss of critical ciliated cells. Consequences of the damage sustained on the fallopian tube epithelium include increased risk of ectopic pregnancy and tubal-factor infertility. Additionally, the resolution of infection can produce new adhesions between internal tissues, which can tear and reform, producing chronic pelvic pain. As a bacterium adapted to life in a human host, the gonococcus presents a challenge to the development of model systems for probing host-microbe interactions. Advances in small-animal models have yielded previously unattainable data on systemic immune responses, but the specificity of *N. gonorrhoeae* for many known (and unknown) host targets remains a constant hurdle. Infections of human volunteers are possible, though they present ethical and logistical challenges, and are necessarily limited to males due to the risk of severe complications in women. It is routine, however, that normal, healthy fallopian tubes are removed in the course of different gynecological surgeries (namely hysterectomy), making the very tissue most consequentially damaged during ascending gonococcal infection available for laboratory research. The study of fallopian tube organ cultures has allowed the opportunity to observe gonococcal biology and immune responses in a complex, multi-layered tissue from a natural host. Forty-five years since the first published example of human fallopian tube being infected *ex vivo* with *N. gonorrhoeae*, we review what modeling infections in human tissue explants has taught us about the gonococcus, what we have learned about the defenses mounted by the human host in the upper female reproductive tract, what other fields have taught us about ciliated and non-ciliated cell development, and ultimately offer suggestions regarding the next generation of model systems to help expand our ability to study gonococcal pathogenesis.

## Introduction

Infections with *Neisseria gonorrhoeae* (gonococcus, GC) most commonly begin at the cervix in females, which marks the dividing line between the lower reproductive tract (vagina, ectocervix) and the upper reproductive tract (uterus, fallopian tubes, ovaries, and endometrium). Cervical infections can be symptomatic or asymptomatic, but without treatment 10–20% of cervical infections ascend to cause infection of the upper female reproductive tract, including the endometrium and fallopian tubes (1). While ascending infection of the fallopian tube may be a dead-end for gonococcal transmission, it is a particularly consequential outcome for the unfortunate host. Fallopian tube infection leads to inflammation (salpingitis) and pelvic inflammatory disease (PID). Following PID, a woman's risk for ectopic pregnancy increases to 9% (from < 2%), tubal-factor infertility increases to 16% (from < 3%) (2), and chronic pelvic pain is experienced by 36% of patients (3). While the proportion of PID cases that are attributable to *N. gonorrhoeae* (< 40%) has fallen relative to *Chlamydia trachomatis* (~60%), gonococcal PID typically presents with more severe symptoms (4). The sharp rise in antibiotic-resistant gonococci raises the risk of reversing gains in preventing gonococcal PID (5).

Unlike many commonly studied bacterial pathogens, *N. gonorrhoeae* is not readily adaptable to laboratory animal models due to its exquisite adaptation to the human host. A female mouse model was developed nearly 20 years ago (6). With refinement in the intervening time, this model has proven very useful, especially in the understanding of complex systemic immune responses model reviewed here (7). Estradiol-treated mice become colonized following intravaginal inoculation and GC can ascend at least as far into the upper reproductive tract as the uterus (8). However, colonization is maintained for only about 10 days and resumption of the murine estrous cycle clears infection (7). Bypassing the vagina via transcervical inoculation allows for transient colonization of the uterus, with successful infection of the majority of animals for up to 24 h. The majority of animals then clear infection by 48 h (9). Despite the success of mouse models, there exist numerous biochemical, physiological, and morphological differences between murine and human female reproductive tracts, as well as between mouse and human immune systems. GC has evolved to exploit human versions of proteins for epithelial cell binding, iron acquisition, and immune evasion, among other features. For modeling human infections, a faithful reproduction of human disease occurs in experimental infection of chimpanzees (10, 11). Studies also can be performed on the infection of human male volunteers. However, both of these models are expensive and not practical for large-scale use. Moreover, human experimental infection necessarily excludes the use of females due to the risk of severe complications. Though the male urethral infection model continues to provide many important insights into host and pathogen biology, this review will focus primarily on modeling infection of the human upper female reproductive tract as the male model has been reviewed elsewhere (12, 13).

As an alternative to animal models for understanding ascending infections and the development of PID, portions of human oviducts (fallopian tubes) can be maintained in culture for days to weeks (14). While pre-menopausal samples are the best for assuring vigorous ciliary activity (15), the hormonal status of donors has no noticeable effect on ciliary activity (16). Therefore, samples obtained from any stage of the menstrual cycle are suitable for use in organ culture. Explants provide an opportunity to study gonococcal infections on a human female epithelial surface that is targeted during natural infection, complete with the complex mixture of ciliated and secretory epithelial cells and multi-layered tissue architecture. This review is intended to summarize what we have learned from fallopian tube organ culture infections with gonococci, what is known about the immunological capabilities of the fallopian tube, and how this immunology relates to our understanding of gonococcal host-pathogen interactions. Lastly, we address how the improvement of human organ and organ-like models are expanding our ability to probe specific molecular and genetic interactions between *N. gonorrhoeae* and the human host.

## Invading the fallopian tube

### Defining a tissue explant infection model for *N. gonorrhoeae*

Prior to the use of human fallopian tube organ cultures as a model ciliated epithelium, tracheal cultures had been used to study infectious agents. Trachea were shown to survive *ex vivo*, displaying ciliary activity for 3–5 weeks (17). In the first published attempt to model gonococcal interactions with ciliated epithelia, fallopian tubes were used alongside tracheal samples from embryonic chicken, cow, human, and adult mouse (16). Non-piliated gonococci were found to grow well on fallopian tube tissue, with an inoculum as low as 10 colony forming units (CFU) reaching a maximal density (10^7^-10^10^ CFU/mL) in 2–4 days. A higher inoculum (10^3^ CFU) reached a maximum density after 1–2 days and caused a complete loss of ciliary activity by 5–6 days. For comparison, uninfected fallopian tube tissue maintained beating cilia for between 14 days and 1 month (or more). Tissue culture media such as Eagle's Minimal Essential Medium (MEM) is sufficient to support tissue survival, though gonococci grow poorly in this media (15). Gonococci will grow, however, in conditioned media from fallopian tube or media supplemented with tissue homogenate. Work that compared rabbit oviducts in culture to human fallopian tube revealed that, similar to the previous work in trachea, the presence of any tissue supports the multiplication of GC in culture. Gonococci, however, neither bind to nor damage rabbit oviduct, and do not cause any reduction in ciliated cell activity (18). Piliated gonococci likewise adhered poorly to the mucosal epithelium of rabbit, pig, cow (19), and guinea pig (20). In all cases, no noticeable histopathology or loss of ciliary activity was observed in animal oviducts compared to human fallopian tubes infected in parallel. One of the seminal observations, made with both piliated and non-piliated strains, is that gonococci bind preferentially to secretory (non-ciliated) cells, though it is the ciliated cells that later die (18, 21). Gonococci are also able to quickly invade the apical side of non-ciliated cells (within ~20 min) (18), and then exit from the basolateral side (transcytosis) but are not observed invading or residing inside ciliated cells (21). When non-pathogenic *Neisseria pharyngis* (now known as *N. cinerea*) was grown in fallopian tube organ culture, the bacteria survived at similar levels to *N. gonorrhoeae*, but failed to elicit any of the decrease in ciliary activity that is characteristic of GC (15), suggesting that human pathogenic *Neisseria* possesses unique factors capable of damaging human female reproductive tract epithelia.

### Epithelial damage and the inflammatory response to gonococci

Early work in the human fallopian tube organ culture model established that GC could colonize explants and recapitulate the damage seen in patients with gonorrhea (22). The specific damage of primary concern is the death of ciliated epithelial cells, which have physiologic functions important in fertility. Loss of the ability of ciliated cells to participate in transporting the fertilized ovum to the uterus is considered a major predisposing factor for tubal factor infertility and ectopic pregnancy (23). In culture, uninfected fallopian tube explants display robust ciliated activity for more than 2 weeks, but tissues infected with non-piliated gonococci show decreases in ciliary activity starting at about 36 h post-infection and exfoliation of ciliated cells starting around 64 h (18). Complete loss of ciliated cell activity occurs by 4–6 days post-infection (16, 18). Multiple groups observed that a decrease in ciliary beat frequency precedes the appearance of visible damage of the epithelial surface (24, 25). The magnitude of reduction in ciliary activity and rapidity of sloughing is more pronounced in infections with piliated gonococci compared to non-piliated, even with equal or fewer CFU recovered from tissues (25).

Ciliated cells are the first to die despite the attachment of gonococci to non-ciliated cells. This observation led to a search for soluble factors (such as toxins) present in filter-sterilized gonococcal broth cultures (cell-free supernatant) and filter-sterilized media from gonococci-infected fallopian tube cultures. Both supernatants were found to contain a heat-stabile, toxic component that is partially removed by adsorption to limulus amoebocyte lysate (LAL), and capable of causing the same ciliated cell death as observed in gonococcal infections (26). This toxic effect was attributed to lipopolysaccharide (LPS), which is present in gonococci as a low-molecular weight version lacking the repeating O-antigen and referred to as lipooligosaccharide (LOS). LOS was purified and shown to decrease ciliary activity at lower concentrations than the microgram quantities measured in infected organ cultures (27). The toxic effect of gonococcal LOS, like damage from gonococcal infection, also appeared to be a human-specific phenomenon, not affecting the ciliary activity of rabbit, pig, or cow oviducts (28). This observation would seem curious now, as LPS/LOS signaling via the eukaryotic immune sensor Toll-like receptor 4 (TLR4) has been characterized as a conserved pathway leading to inflammatory responses in all of the above species (29, 30). Adsorption of LOS with LAL, however, did not eliminate all of the soluble toxic activity produced by gonococci, implicating another factor(s) causing toxicity. Around the same time, it was just beginning to be recognized that gonococci release soluble fragments of peptidoglycan (PG), in particular monomeric fragments consisting of *N*-acetylmuramic acid and *N*-acetylglucosamine sugars linked to three- or four-amino acid long peptide chains (with 80% as tripeptide, 20% as tetrapeptide) (31, 32). Treatment of organ cultures with isolated PG monomers, reduces ciliary activity and causes death and sloughing of ciliated cells (33). The later isolation from *Bordetella pertussis* of tracheal cytotoxin (TCT), a tetrapeptide PG monomer identical to the minority monomer species released by gonococci, reinforced the idea that small PG monomers can cause ciliated cell cytotoxicity, in this case in the trachea of hamsters (34, 35). The further discovery that PG is found as a contaminant in crudely purified LPS preparations (36), and the recognition that PG monomers are sensed differently by the same receptor in different species (37), implicates PG as a major mediator of species-specific oviduct toxicity. Certainly, both LOS and PG are toxic products, abundantly released from growing gonococci and capable of contributing individually to inflammation. Together, it is likely they have an even greater impact, as PG monomers are known to synergize with LPS to provoke a larger host response than either alone (38).

**Figure 1:**
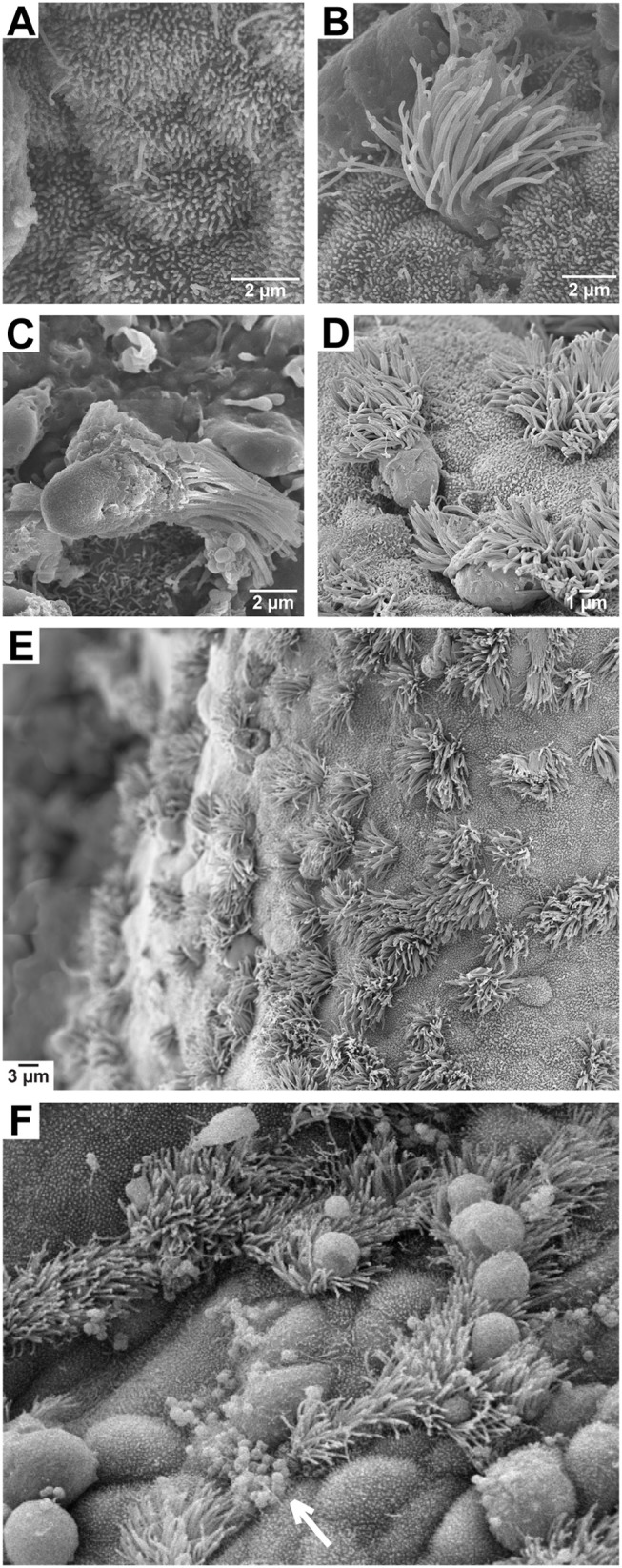
Scanning electron micrographs of fallopian tube explants. **(A)** Non-ciliated (secretory) epithelial cell; **(B)** ciliated epithelial cell; **(C)** ciliated epithelial cell sloughed during gonococcal infection; **(D)** ciliated cells sloughing following a 24 h treatment with 2 ng/mL TNFα; **(E)** an untreated epithelial layer displaying normal cell morphology; **(F)** an epithelial layer during gonococcal infection showing binding of most bacteria to non-ciliated cell surfaces (arrow) and swelling of cells that precedes ciliated cell sloughing.

While gonococcal LOS and PG fragments are ascribed causative roles in epithelial damage, neither is a “toxin” in the same sense as a lytic pore-forming toxin or a compound capable of poisoning cellular processes to create a direct toxic effect. Rather, LOS and PG are recognized by the host as pathogen-associated molecular patterns (PAMPs) and induce programmed defense responses. One of the first host inflammatory responses measured from gonococcal infection of fallopian tube organ cultures was the induction of tumor necrosis factor (TNF) (39). Addition of TNFα to fallopian tube explants reduces ciliary activity in a dose-dependent manner, and reproduces the characteristic death and sloughing of ciliated cells (Figure 1). Rising concentrations of TNFα during gonococcal infection correlate linearly with a decrease in ciliary activity (40). In the analogous *B. pertussis* system, it should be noted that interleukin (IL) 1 (and not TNFα) was implicated as the host factor driving respiratory epithelial damage. IL-1 addition was shown to be capable of inducing ciliated cell death similar to that seen in TCT treatment of hamster tracheal rings (41). Though the host organism and infection system are different, the role of IL-1 is noteworthy as this cytokine is also implicated as the driver of epithelial damage in *Chlamydia trachomatis* infections of human fallopian tube organ cultures (42).

Gonococcal infection is capable of inducing transcription of numerous cytokines and chemokines in epithelial cell lines such as ME-180, HeLa, and HaCaT via activation of the transcription factor nuclear factor kappa-B (NF-κB) (43). In fallopian tube explants, TNFα and IL-1β, as well as the inflammatory cytokines/chemokines IL-6, monocyte chemoattractant protein-1 (MCP-1, CCL2), macrophage inflammatory protein-1β (MIP-1β,CCL4), and granulocyte-macrophage colony-stimulating factor (GM-CSF) are made in response to gonococcal infection and detected by enzyme-linked immunosorbent assay (ELISA) after as little as 3 h (44, 45). While levels of TNFα, IL-1β, IL-6, and MCP-1 seem to generally increase out to 24 h, MIP-1β and GM-CSF appear to peak at 12 h and decline by 24 h. The kinetics of some cytokine responses were shown to vary between piliated and non-piliated variants, with cytokine induction by piliated gonococci potentially delayed by several hours (45). Since the vast majority of gonococci recovered from symptomatic natural infections are piliated (46, 47), a delay in cytokine induction in fallopian tube explants may indicate a role for pili in suppressing certain immune responses during ascending infection.

Nitric oxide (NO) is another component of the inflammatory host response that is produced on mucosal epithelia, in particular NO made by inducible nitric oxide synthase (iNOS, encoded by *nos2*). In hamster tracheal epithelial (HTE) cells, TCT and IL-1 were shown to induce nitrite production (a proxy for NO), and applying nitric oxide synthase inhibitors prevented the loss of ciliary activity in hamster tracheal rings treated with TCT (48). In the hamster trachea, NO production localizes only to the non-ciliated (secretory) epithelial cells (49). Though intriguing, this finding regarding the distribution of NO production has not been reproduced in humans or in reproductive tract tissues. During gonococcal infection of fallopian tube organ cultures, iNOS transcription is induced ~35-fold, but treatment of uninfected fallopian tube tissues with the NO donor *S*-Nitroso-*N*-acetylpenicillamine (SNAP) did not induce cellular damage as measured by lactate dehydrogenase (LDH) release (50). Treatment of infected tissues with iNOS inhibitors also produced mixed results in reducing cellular damage (50). The inhibition of fallopian tube damage that was seen during treatment with N(G)-Nitro-L-arginine methyl ester (L-NAME) corresponded with a reduction in bacterial CFU. A decrease in bacterial proliferation presumably accounted for a corresponding reduction in tissue damage. Interestingly, a reduction in viable gonococci during inhibition of iNOS aligns with data that iNOS activity increases gonococcal survival within primary human cervical epithelial (pex) cells (51). While it is unclear whether NO participates directly in the damage observed in the fallopian tube during gonococcal infections, GC has adapted methods to avoid killing and perhaps even exploit NO during host colonization.

Matrix metalloproteinases (MMPs) are host enzymes that function in the degradation of extracellular matrix, disassembly of cellular junctions (including for immune cell infiltration), and activation (by cleavage) of cytokines and growth factors (52). Numerous bacteria or bacterial products (including PG and LPS) have been shown to induce various MMPs (including MMP1, 2, 3, 7, 9, 10, and 11) (53–56). When bacteria induce MMPs, host tissue can sustain collateral damage, such as when Gram-positive *Enterococcus faecalis* activates MMP9, contributing to adverse outcomes of surgical wound infections (57). During gonococcal infection of fallopian tube epithelial cells, MMP2 accumulates intracellularly while MMP9 secretion is increased (MMP3 and MMP8 levels are unchanged) (58). In fallopian tube explants, MMP8 expression is increased following GC infection, while transcription of MMP3 and MMP9 (as well as the TIMP metallopeptidase inhibitor 1, TIMP-1) are unchanged (59). The functions and localization of MMPs, when coupled with data indicating their induction during fallopian tube infection, makes these enzymes prime candidates for mechanistic involvement in epithelial damage. In cell culture, gonococci cause disruption of E-cadherin and β-catenin from adherens junctions between fallopian tube epithelial cells (60). GC also disrupt the apical junctions of polarized HEC-1-B (endometrial) and T84 (colonic epithelial) cells by activating epidermal growth factor receptor (EGFR) signaling (which also involves redistribution of β-catenin) (61). The disassembly of apical junctions is also seen in an endocervical tissue model of infection, where GC activates intracellular non-muscle myosin II and induces calcium flux to promote the exfoliation of columnar epithelial cells (62). Additional investigation is needed into the composition of cellular junctions between ciliated and non-ciliated fallopian tube epithelial cells, the activity of bacterially-induced MMPs, and the intracellular signaling pathways activated by gonococci to determine what factors make ciliated cells especially sensitive to exfoliation.

The exact mechanisms by which ciliated cells die and are removed from the epithelial layer remain unknown. Morphologically, cells do not appear to lyse as would be expected from necrosis or pyroptosis (though IL-1β is released during infections). In cultured human fallopian tube epithelial cells, GC inoculated at various multiplicities of infection (MOI = 1, 10, or 100) all result in production of TNFα, which can cause apoptosis. However, only the lowest MOI showed significant Caspase-3 activation (indicative of apoptotic cell death) (63). Since TNFα addition alone can induce apoptosis and addition of more GC (higher MOI) blocks the TNFα-dependent apoptosis, it can be hypothesized that GC contact or some GC-produced soluble factor produces an anti-apoptotic effect. Indeed, GC is known to increase the expression of anti-apoptotic factors in human urethral epithelial cells (64, 65), and human End1 endocervical epithelial cells (66). GC also confers protection from apoptosis in neutrophils (67, 68). However, GC also induces pro-apoptotic genes in HeLa cells (69). The outer membrane porin PorB has been implicated in induction of apoptosis by (1) trafficking to mitochondria in murine bone marrow-derived macrophages (BMDMs) and human THP-1 macrophages, and (2) initiating the intrinsic pathway of apoptosis in mouse BMDMs (70). During fallopian tube explant infections, GC were more often seen contacting (or in close proximity to) non-ciliated cells, which could facilitate the induction of a protective response. Ultimately, a better understanding is needed of the differences between ciliated and non-ciliated cells in their ability to sense and respond to TNFα and other components of the host defense response.

### Factors conferring adherence to fallopian tube epithelia

Adhering to host surfaces, whether to facilitate physical anchoring for replication or to induce uptake into host cells, is a critical step in the life cycle of many bacterial pathogens. Such is the case for GC, which can multiply extracellularly on mucosal surfaces, or proceed through a stepwise process of first binding tightly to columnar epithelial cells, being endocytosed, transiting through epithelial cells, and exiting into the subepithelial space (71, 72). Early observations of *N. gonorrhoeae* adherence during human fallopian tube organ culture infection focused on the two best known (and most easily observable) mediators of adherence: pili and opacity (Opa) proteins. Piliated GC bind to (and damage) the mucosal surface more rapidly than non-piliated variants of the same strain (25). Piliated gonococci were also observed binding to the tips and surfaces of microvilli on non-ciliated cells (73), a phenomenon later observed in infections of cultured HEC-1-B endometrial carcinoma cells as a precursor to cellular invasion (74). The exact receptor that gonococcal pili engage during fallopian tube infection for either adherence or invasion is unknown. Both CR3 (a CD11b/CD18 integrin heterodimer) and CD46 (MCP) have been implicated as ligands for pili (75, 76), and both are found on the fallopian tube epithelium (77, 78). Expression of CR3, however, decreases progressively in ascending tissues, reaching its lowest relative level in the fallopian tube (77). In addition, questions remain as to whether the interaction of pili with CD46 functions to mediate adherence or rather a downstream intracellular signaling event (79–82). In the endometrium, carcinoembryonic antigen-related cell adhesion molecules (CEACAMs/CD66), asialoglycoprotein receptor (ASGP-R), and CR3 have each been implicated in gonococcal adherence (83).

Gonococci can still bind to and damage fallopian tube epithelia through pilus-independent mechanisms (18), and Opa proteins alone are sufficient to confer adherence to fallopian tube epithelium (84). In the only study to look exclusively at the role of Opa proteins in attachment to fallopian tube, Opa- variants of F62-SF were found to bind exclusively to non-ciliated cells (as observed previously) as did OpaC+ and OpaD+ strains (which bound to microvilli). Strains expressing OpaB, however, were seen on both ciliated and non-ciliated cells, with OpaA-expressing strains binding to ciliated cells in large clumps (85). Interestingly, the OpaC and OpaD-expressing variants were judged the most damaging to epithelia while OpaA-expressing were the least damaging. Despite Opa proteins being well characterized as adhesins and routinely associated with successful human infections (86), early work in fallopian tube organ culture suggested that piliated and transparent (Opa-) bacteria bind better (by 10–100x) to fallopian tube explants than piliated and opaque (Opa+) bacteria (22, 73). In laparoscopic sampling from the fallopian tubes of women with salpingitis, the same group recovered predominantly GC with transparent colony phenotypes (>95% Opa-) while endocervical isolates were 50% or greater Opa+ (87). The majority of Opa proteins (which vary in number between gonococcal strains) bind to distinct CEACAMs, though at least one Opa binds instead to heparin-sulfate proteoglycans (HSPG) (88). In a study of low-passage isolates of GC from cervix and male urethra, the majority of strains bound to transfected Lec11 cells (a CHO derivative) stably expressing CEACAM1, CEACAM5, or CEACAM6. Fewer showed robust binding to CEACAM3 and very few bound CEACAM8 (89). Human CEACAM5, when expressed in mice, also contributes to increased recovery of GC during murine lower genital tract infection (90). The expression levels of particular CEACAMs on cells of the human female genital tract has been questioned, however, with evidence that CEACAM1, 3, 5 (CEA), 6, and 8 are not highly expressed on primary fallopian tube, primary cervix, HEC-1-B endometrial cells, or HeLa cervical carcinoma cells (91). It is not known which CEACAM (or HSPG) is the most available target of Opa proteins in the fallopian tube, nor is it known what threshold level of expression is needed for Opa-CEACAM binding in any given tissue. It is clear that Opa proteins facilitate adherence to cultured epithelial cells and contribute to bacterial fitness during successful colonization of the murine lower genital tract (92). In primary human ectocervical and endocervical cells, however, the absence of Opa expression does not decrease adherence (76). It is still possible that Opa proteins contribute to the mix of adhesins needed to address the changing receptor availability and immunological context encountered during the progression of ascending from the endocervix to the fallopian tube (83). In a study of seven patients with acute salpingitis (fallopian tube inflammation), laparoscopic isolation of bacteria from fallopian tube and cul-du-sac revealed a higher proportion of Opa- than Opa+ in these tissues (22). The laparoscopy samples also had a consistently higher proportion of Opa- GC than matched cervical isolates from the same patient. These data suggest that whatever factors are driving Opa protein production at the endocervix, Opa proteins may be turned off or selected against when bacteria are infecting the fallopian tube.

Gonococcal lipooligosaccharide has already been discussed as an important trigger of host inflammatory responses, but LOS on the cell surface may also function in bacterial binding and invasion of fallopian tube epithelial cells. Several different gonococcal LOS variants bind to Galectin-3, a β-galactoside-binding protein expressed on non-ciliated cells of fallopian tube epithelium as well as on HEC-1-B endometrial adenocarcinoma and PC3 human prostate adenocarcinoma cells (93). The “triggering receptor expressed on myeloid cell-2” (TREM-2) was also identified as a ligand for gonococcal LOS, and is constitutively expressed on human fallopian tube epithelium, ME-180 and HeLa cervical carcinoma cells, ectocervical Ect1/E6E7 cells, endocervical End1/E6E7 cells, vaginal Vk2/E6E7 cells, and THP-1 monocytic cells (94). The resident microbiota in the female lower reproductive tract contribute sialidase activity that processes the terminal sialylation present on LOS, a process that has been implicated in promoting transmission to males by unmasking a terminal galactose that binds to ASGP-R on urethral epithelial cells (95, 96). It is unclear how the sialylation state of LOS impacts the pathogenesis of fallopian tube infection. Increasing LOS sialylation is known to decrease Opa-dependent invasion of Chang conjunctival and ME-180 cervical epithelial cells (97). Several studies also report rapid internalization of gonococci into fallopian tube secretory epithelial cells (21, 22), perhaps implicating a low level of LOS sialylation during ascending infection.

Natural hormonal cycling likely plays a role in the expression of gonococcal factors important for adhesion, immune evasion and virulence. Before specific Opa proteins were recognized as the factors determining colony opacity/transparency, variations in the visual appearance of isolates were noticed to change (along with the likelihood of recovering viable gonococci) based on the stage of the menstrual cycle (47). Isolates obtained in close temporal proximity to menstruation were found to lack the surface proteins associated with opacity. The overall variation in recovery phenotypes during the menstrual cycle was attributed to the action of progesterone (or substances acting similarly to progesterone). While progesterone has been investigated for its ability to enhance survival of gonococci in primary cervical epithelial (pex) cells (51), its role in ascending infection is unknown. However, since progesterone causes a significant lowering of ciliary beat frequency in fallopian tube (98), progesterone could reasonably be expected to have some impact on gonococcal adherence or colonization in this tissue. Additionally, fallopian tube tissue from users of both hormonal and non-hormonal (copper intrauterine device) contraception display changes in the surface expression of CD46 and the HSPG syndecan-1, but not CD66 (CEACAM) (78). Much remains to be determined about how bacteria, adherent at the cervix, transit the uterus to enter the fallopian tube. It is likely that gonococci have adapted to utilize numerous host receptors in sequence (83), and are able to adjust accordingly to whatever tissue environment they find themselves in as they make their way into the upper female reproductive tract.

### Insights from *Chlamydia trachomatis* infections in fallopian tube explants

*Chlamydia trachomatis* (Ct) is the most commonly reported sexually transmitted infection (STI) and another bacterium that can ascend to the fallopian tubes, causing PID and tubal-factor infertility. Fallopian tube organ culture explants have also been used to study the pathogenesis of Ct, with early observations in this system noting that Ct is able to bind to and infect both ciliated and non-ciliated cells (99). Unlike GC infection, ciliated cell function did not appear to be affected by Ct, though microvilli were lost on infected non-ciliated cells, which became rounded and lost attachment to neighboring cells. By 72 h post-infection, ruptured cells could be observed, characteristic of the cell lysis that results from release of infectious elementary bodies (EBs) at the end of the Ct intracellular life cycle. The observed loss of cellular polarity and disruption of cell-cell junctions in Ct-infected fallopian tube is accompanied by β-catenin recruitment from adherens junctions to Chlamydial inclusions (100). While disassembly of junctions likely contributes directly to epithelial disruption, β-catenin is also tightly regulated at the cellular level as a component of the developmentally important Wnt signaling pathway. Increased Wnt signaling during Ct infection induces numerous changes in fallopian tube tissue, including up-regulation of the stem-cell marker Olfactomedin 4 (OLFM4) (100), which is a target of NF-κB and Notch signaling pathways. OLFM4 also participates in the curtailing of innate immune responses by negatively regulating nucleotide-binding oligomerization domain-containing protein 1 (NOD1)- and NOD2-dependent NF-κB activation (101).

Cultured epithelial cell lines (HeLa, SiHa, HT-29, SW620, and primary endocervical) infected with Ct secrete proinflammatory cytokines (IL-8, IL-6, GM-CSF, and GROα/CXCL1), but cytokine secretion is delayed until 20–24 h post-infection, after lysing cells release IL-1α (102). Blocking IL-1α blunts the cytokine burst, suggesting that neighboring epithelial cells are sensing IL-1α and initiating an amplification of the inflammatory response. In fallopian tube organ cultures, addition of exogenous IL-1α was shown by scanning electron microscopy (SEM) and histological staining to cause damage similar to Ct infection, with IL-1 receptor antagonist (IL-1RA) and p38 mitogen-activated protein (MAP)-kinase blockade (downstream of IL-1 sensing) reducing epithelial damage (42). The ability of cytokines (TNFα in the case of GC, IL-1α for Ct) to exacerbate immune-driven pathology is common to both infections, as is potentially the role of matrix metalloproteinases (MMPs) in tissue disruption. Transcriptional analysis of Ct infecting monolayers of HEp-2 cells revealed induction of MMP2 and MMP9 (103), the same MMPs with altered expression during GC infection of fallopian tube (58). Though Ct and GC have different lifestyles that bring them into conflict with the host in different ways, both organisms induce a pro-inflammatory response in the fallopian tube epithelium, where the long-term sequelae of infection appear to be the result of host-driven inflammatory pathology.

## Defending the fallopian tube

### Pattern recognition receptors in the innate immune response

Colonization of GC in the upper female reproductive tract (FRT) involves bacteria binding to and interacting with epithelial cells. Epithelial cells are ready to respond to the presence of invading pathogens through the expression of a variety of microbial pattern recognition receptors (PRRs). Surveys of PRR expression throughout the FRT revealed expression of all identified toll-like receptors (TLR1-10), as well as cytosolic sensors NOD1, NOD2, retinoic acid-inducible gene I (RIG-I) and melanoma differentiation-associated protein 5 (MDA5) (104–107). In general, receptor expression appears to be constitutive and nearly ubiquitous across different regions of the FRT, but notable differences exist in the levels of receptor expression between tissues. TLR2 transcripts are higher in the fallopian tube and cervix than other FRT sites, while TLR4 transcript is higher in the fallopian tube and endometrium with lower expression in the lower tract (104). Immunohistochemical staining confirmed the presence of TLR1-3 and 5-6 throughout the FRT, and indicated detectable TLR4 only in the fallopian tubes, uterus, endometrium, and endocervix (TLR7-10 were not tested in this work) (105). The absence of TLR4 from the ectocervix and vagina would presumably reduce the triggering of inflammatory signaling that may be disadvantageous for maintaining a healthy vaginal commensal community. Transcript for TLR10 is perhaps the most restricted, having first been reported in the endometrium (108). That finding was disputed by later work that surveyed the entire FRT and found detectable expression only in the fallopian tube (106). Differences in detection may be due in part to variations in expression that occur at different points in the menstrual cycle reviewed in (109). A high relative expression of TLR10 in the fallopian tube is particularly interesting since TLR10 appears to act as an anti-inflammatory TLR. TLR10 heterodimerizes with TLR2 (which also has high expression in the fallopian tube) to inhibit inflammatory signaling and promote the production of anti-inflammatory IL-1 receptor antagonist (IL-1Ra) (110). The presence of TLR10 may then serve as a check on out-of-control inflammation in the Fallopian tube. The localization of TLRs can also be further restricted (or enriched) at the cellular level within the fallopian tube. Though TLR4 expression was reported to be generally high in fallopian tube, this receptor may be preferentially present on the surface of oviductal stromal fibroblasts, underneath the epithelial layer, and not on the epithelial cells themselves (111). The absence of TLR4 on epithelial cells could function to protect against the spurious activation of pathologic inflammation in the fallopian tube. However, the demonstrated ability of GC to disrupt epithelial integrity and invade through the epithelial layer (18, 21) likely puts the bacterium in proximity to TLR4 on fibroblasts. Some evidence also exists that ciliated and non-ciliated epithelial cells express TLRs at different levels, with ciliated cells expressing all 10 TLRs at higher levels than non-ciliated cells (112). It is unclear as of yet how differential responsiveness to TLR agonists might relate to the different cell fates seen during GC infections.

The cytosolic PRRs NOD1 and NOD2 recognize various fragments of PG (113), which are shed in abundance by gonococci and known to cause damage during fallopian tube infection (33). Synthetic muramyl peptides, some of which are similar to those naturally released by gonococci, can be endocytosed by HEK293T cells to activate NOD1 and NOD2 (114). When the fallopian tube, endometrium, endocervix, and ectocervix regions were surveyed for NOD receptor expression, all tissues were found to express NOD1, NOD2, and the adaptor receptor interacting serine/threonine kinase 2 (RIPK2) (106). Fallopian tube was shown to have the highest relative expression of both NOD1 and NOD2 compared to other FRT tissues (107). Primary fallopian tube epithelial cells were also shown to be capable of generating an IL-8/CXCL8 response to the addition of the NOD1 agonist D-gamma-Glu-*meso*-diaminopimelic acid (iE-DAP) and the NOD2 agonist muramyl dipeptide (MDP) (106). Fallopian tube organ cultures treated with conditioned media from *N. gonorrhoeae* or *N. meningitidis* broth cultures both induce an IL-8 response proportional to the release of human NOD1 agonist (115).

Gonococcal products, whether on the bacterial surface, secreted, or delivered by membrane vesicles, have been shown to interact with several TLRs and NOD receptors, which likely initiate the early innate immune signaling during ascending infection. Cell-free supernatant and gonococcal lysates (containing whole PG sacculi) activate NF-κB via human NOD1 and NOD2 (116). The NOD1 activation is dependent upon the periplasmic conversion of a gonococcal cell wall rich in tetrapeptide-stem PG into soluble fragments with primarily tripeptide stems that are agonists for human NOD1 (117). Gonococci can activate human NOD2 with released glycosidically-linked peptidoglycan dimers, and with multimeric PG (as might result from cell lysis) that is converted by host lysozyme to produce reducing disaccharide-containing PG fragments (118). Gonococcal lipooligosaccharide activates TLR4 signaling, including both the MyD88 pathway that activates NF-κB and the interferon regulatory factor 3 (IRF3) pathway that activates Type I interferons (119). The acylation state of the lipid A molecule is critical to gonococcal TLR4 activation, with the wild-type hexa-acylated version required for TLR4-dependent signaling (120). The placement of various sialic acid moieties (sialylation) on LOS appears not to influence TLR4 activation (121), while the presence of phosphoryl substituents on the lipid A molecule does influence TLR4 activation (119). Pathogenic *Neisseria* are potent activators of TLR4 signaling in part because they are prone to membrane blebbing, or the release of outer membrane vesicles, which functions to spread LOS, membrane proteins and associated intracellular cargo out from the cell, increasing the potential for bacterial products to contact PRRs (122–124).

Despite the lack of TLR4 expression in certain regions of the FRT, and a confirmed lack of expression in isolated vaginal, ectocervical and endocervical cells lines, GC is still able to activate NF-κB and induce proinflammatory cytokines in End-1 endocervical cells in the absence of TLR4 (125). TLR2 has long been recognized as another PRR activated by pathogenic *Neisseria*, an activity that has been largely attributed to recognition of the major outer membrane porin (PorB) (126, 127). Most studies of porin-TLR2 interaction have involved PorB from *N. meningitidis*, which is considered a stronger TLR2 agonist than *N. lactamica* PorB, which has been investigated for its vaccine adjuvant potential (128, 129). Gonococcal PorB, however, has recently been shown to have low TLR2 agonist activity when protein is refolded during purification, and higher TLR2 agonist activity when PorB is unfolded or aggregated (130). The outer membrane lipoprotein, Lip (also known as H.8 antigen) from GC has been shown to activate an NF-κB reporter in HEK293 cells expressing TLR2, and induce IL-6 and IL-8 from End-1 endocervical epithelial cells (131), confirming that GC possesses multiple potential TLR2 agonists.

Gonococci are able to activate additional cellular surveillance pathways, in particular activation of TRAF-interacting protein with forkhead-associated domain (TIFA) (132), and cyclic-GMP-AMP synthase (cGAS) (133). TIFA-dependent signaling is initiated by the intracellular detection of heptose-1,7-bisphosphate (HBP), a byproduct of gonococcal LOS production. Though a role for HBP has not yet been explored in fallopian tube infections, gonococcal supernatant containing HBP induces inflammatory responses in cultured End-1 endocervical epithelial cells, human macrophages, and human neutrophils (132). HBP has been shown in *Helicobacter pylori* infections to produce an earlier NF-κB response than NOD1 agonist (134). The cGAS enzyme is activated by detection of cytosolic double-stranded DNA (dsDNA) and initiates STING/TBK-1/IRF3 activation leading to induction of type I interferon. During GC infection of human monocytes (THP-1 cells), TLR4 and cGAS synergize to induce production of interferon-β (IFN-β) (133). Even with this observation, it is uncertain what role IFN-α or IFN-β has during gonococcal infection, since IFN-β can be both beneficial and detrimental to the host in clearing infection. In addition, the source of cytosolic gonococcal dsDNA sensed by cGAS remains uncertain, though presumed to originate from spontaneous bacteriolysis. It is therefore plausible that the proliferation of extracellular bacteria releasing TLR4 agonist, while some bacteria are phagocytosed and lyse inside responding macrophages, creates a scenario for increased IFN-β production during fallopian tube infection.

### Immune cells in fallopian tube surveillance

Like other mucosal surfaces, the fallopian tube contains a complement of resident immune cells that can be found within the epithelial layer and just behind the epithelium in the lamina propria. A flow cytometry-based survey of the entire human female reproductive tract across 28 patients ranging from 26 to 66 years old revealed 6–20% of the total cellular content to be leukocytes (CD45^+^), with fallopian tube and uterus containing a higher proportion of immune cells than the cervix or vagina (135). Across all tissues, 30–60% of the leukocytes were T cells (CD3^+^), with granulocytes (CD66b^+^, likely polymorphonuclear leukocytes, PMNs) as the major population observed in the fallopian tube. However, a later study that focused exclusively on the fallopian tube epithelium and utilized both flow-cytometry and immunohistochemistry with an updated and expanded set of cellular markers reached a somewhat different conclusion on leukocyte distributions (136). Ardighieri et al. (136) surveyed 10 patients (5 pre-menopausal, 26-35; 5 post-menopausal, 63-73) and observed macrophages (CD68^+^ CD163^+^) and dendritic cells (DCs) (CD11c^+^) as the most abundant innate immune cells present in the fallopian tube, at ratios (to total nucleated cells) of ~1/30 and 1/21, respectively. While all combinations of CD11c^+/−^ and CD163^+/−^ cells (overlapping macrophage and DC markers) were present, CD11c^+^ CD163^−^ DCs were abundant. These cells were observed at regular intervals, with cell bodies in the basal lamina and long intraepithelial projections facing toward the lumen. CD11c^+^ CD163^+^ cells were the most abundant population in the lamina propria and muscle wall (under the epithelium). In this same analysis (136), neutrophils were noted as a minor population localized almost exclusively intravascularly and present only in the lamina propria and muscle wall, the same localization as minor populations of mast cells (CD117^+^), plasmacytoid DCs (CD303^+^), plasma cells (CD138^+^), and regulatory T cells (CD3^+^Foxp3^+^). Whether or not neutrophils are a major population in the normal fallopian tube, these cells are well characterized as important components of the cellular response to GC infections. Neutrophils migrate rapidly from the bloodstream to sites of tissue inflammation and are found in high numbers in gonococcal infection of the male urethra and female cervix. As the role of neutrophils in GC infection of the female reproductive tract has recently been reviewed (137), the following section will focus on other fallopian tube-resident immune cell types and the outcomes of their potential interactions with gonococci.

Dendritic cells are critical front-line phagocytes and potent antigen-presenting cells, which function in the activation of T cells (138). DCs that encounter antigen in the presence of proinflammatory signals are induced to produce IL-12 and costimulatory molecules that encourage the development of T helper cells. Contact with GC, however, has been shown to induce expression of anti-inflammatory molecules (IL-10, PD-L1) from human and murine DCs, and both human and murine DCs exposed to GC are less able to stimulate proliferation of CD4^+^ T cells (139, 140). Porin (PorB) delivered via outer membrane vesicles (OMVs), has been shown to contribute to the suppressive effect of GC on DCs, in spite of any ability of porin to engage stimulatory TLR2 signaling (130). Though the mechanics of GC suppression of DC activity are still incompletely understood, several potentially important receptor-ligand interactions have been identified. Gonococcal LOS binds to C-type lectin receptors on DCs, with variations in glycosylation influencing which receptor is engaged (141). Wild type LOS from strain F62 with terminal *N*-acetylgalactosamine (GalNAc) binds to “macrophage galactose-type lectin” (MGL, also present on DCs). An *lgtD* mutant with terminal galactose is recognized through an unknown receptor. While an *lgtB* mutant with terminal *N*-acetylglucosamine (GlcNAc) binds to dendritic cell-specific intercellular adhesion molecule-3-grabbing non-integrin (DC-SIGN). The *lgtD* and *lgtB* deletions mirror the phenotypes that occur naturally due to slipped-strand mispairing. All of these variants have the ability to stimulate DCs to activate T cells, although LOS with a terminal GalNAc induces less IL-10 than other variants. This finding suggests that interaction with DCs may exert selective pressure for GC to vary LOS during host infection. Opa proteins, through their engagement of CEACAM1, have been shown to reduce the ability of DCs to stimulate memory responses from T cells against human immunodeficiency virus-1 (HIV-1), in part through down-regulation of the costimulatory molecule CD83 (140). It should be noted, however, that other groups have previously observed up-regulation of CD83 (and major histocompatibility complex class I, MHC-I) on DCs exposed to GC, as part of investigating how GC promotes HIV-1 infection of DCs (142). The interaction of GC with DCs is particularly interesting since DCs are critical target cells for HIV-1 infection (both pathogens share DC-SIGN as a receptor). Infections with GC have been shown to create an increased risk for HIV-1 infection through multifactorial mechanisms that remain unclear (143).

Macrophages, the next most abundant immune cell type in the fallopian tube, are primary phagocytes that are likely to encounter GC that has invaded through or between epithelial cells and entered the submucosa. Fallopian tube explants release cytokines and chemokines capable of recruiting macrophages during gonococcal infection, such as MCP-1 (CCL2) and MIP-1β (CCL4) (45). Macrophages exposed to GC produce proinflammatory cytokines such as TNFα, IL-1β, IL-6, MCP-1 (CCL2), MIP-1α (CCL3), MIP-1β (CCL4), RANTES (CCL5), GROα (CXCL1), and CXCL10, which also likely promote PMN and T cell recruitment (144–146). Macrophages are able to phagocytose GC, with *in vitro* infections of human peripheral blood mononuclear cells (PBMCs) resulting in near complete internalization within 1 h of exposure and the killing of >60% of internalized bacteria in that same period (147). Similar results were reported for THP-1 monocytes, with >60% killing by 2 h post-phagocytosis and >80% by 5 h (146). Despite rapid eradication of the majority of the population inside PBMCs, by 6 h post-infection GC are seen persisting in macrophages. Survival occurs, in part, through the activity of the lipoprotein Ng-MIP (in the macrophage infectivity potentiator family of peptidyl-prolyl cis/trans isomerases) and by gonococcal manipulation of host gene expression to acquire necessary iron while inside (or in close association with) macrophages (146, 147). Gonococci that have been phagocytosed by macrophages are then capable of manipulating or inducing various cell death pathways. In human monocyte-derived macrophages (MDMs), intracellular gonococci are associated with increased macrophage death, with infection inducing caspase 1 and 4, indicative of pro-inflammatory (pyroptotic) cell death (148). Both THP-1 monocytes and PMBCs activate the NLRP3 inflammasome, promoting rapid cell death via pyronecrosis (145). In cultured macrophage lines, GC strain FA1090 protects U937 macrophages from staurosporine-induced apoptosis (anti-apoptotic activity), though infection induces apoptosis in the THP-1 cell line (149). Interestingly, U937 and THP-1 cell lines expressed different cytokine profiles during GC infection. For example, THP-1 cells secreted more IL-1α and IL-6, while U937 secreted more IL-10, an indication that these cell lines may have different pre-existing set points on the continuum of macrophage polarization states (150). Polarization of macrophages may itself be a relevant occurrence during infections, as primary human MDMs have been shown to polarize toward an M2 (specifically M2b) phenotype during infection with piliated, Opa^+^ gonococci. This is despite the fact that cytokines expressed by these macrophages include classically M1 and M2 molecules (matching the cytokines mentioned above) (151). A similar promotion of a tolerogenic phenotype (up-regulation of IL-10 and transformaing growth factor- β, TGF-β) is observed in infections of mouse macrophages (152). Increased expression of the immunosuppressive molecule PD-L1 on human MDMs along with a reduced ability of GC-infected MDMs to stimulate CD4^+^ T cells (151), mirrors the observations from DCs (139, 140). GC is therefore capable of manipulating both of these antigen-presenting cell types to discourage a productive adaptive immune response.

The major point of agreement between the two studies that estimated fallopian tube leukocytes is the abundance of T cells in this tissue (135, 136). In particular, the later study specifies cytotoxic (CD8^+^) T cells as the overwhelming majority of the CD3^+^ population in the intraepithelial compartment (1/15 ratio of CD8^+^ cells to epithelial cells). By contrast, gamma delta (TCRγδ^+^) T cells were observed at 1/120 and T helper (CD4^+^) cells were observed at only 1/400 in the intraepithelial space. Natural killer T (NKT) cells with invariant Vα24-JαQ TCRα were absent. Little to nothing is known about the role of T cells in the human response to gonococci in the fallopian tube, rather, most of what is known about T cells during gonococcal infection has come from studies of mouse models of intravaginal infection. During mouse infection, GC induces cytokines that indicate the presence of a T helper 17 (Th17) cell response (including the cytokine IL-17A) and not the cytokines expected from a Th1 response (153). In the same work, isolated mouse PBMCs and the human THP-1 cell line were both shown to make Th17-associated cytokines in response to GC infection or OMV treatment. Interestingly, blocking IL-17A or knocking out the receptor in mice, decreased neutrophil recruitment, increased recovered CFUs, and prolonged infection. These results indicate a disruption in an otherwise productive response that could clear gonococcal infection. One mechanism by which gonococci induce a Th17 response is through TLR4 recognition of gonococcal LOS, as Th17 responses are diminished in TLR4-mutant mice (154). Opa proteins then act to influence the Th17/Th1 balance, suppressing Th1 and Th2 responses (155). Central to determining the nature of the host T cell response, is the induction of TGF-β, IL-10, and type 1 regulatory T cells during gonococcal infection, which together promote Th17 responses and suppresses Th1 and Th2 responses (155, 156). Regulatory T cells (T_regs_), including both TGF-β1-positive CD4^+^ cells and CD4^+^ CD25^+^ FoxP3^+^ cells, had been previously observed following mouse intravaginal infections (8). T_regs_ increase in number in the mouse genital tract draining lymph nodes following intravaginal gonococcal infections and TGF-β1^+^ CD4^+^ cells are observed in the mouse uterus. Though mice represent an imperfect proxy for human ascending infection and PID, the mouse model has provided valuable insight into the possible mechanistic basis of key observations in humans. Namely, why people are unable to mount a protective memory T cell response to a noticeably inflammatory bacterial infection. Future work is needed to determine the location of specific T cell subsets and their activation state during infections modeled in human tissue or *in situ* during or following natural infections.

**Figure 2:**
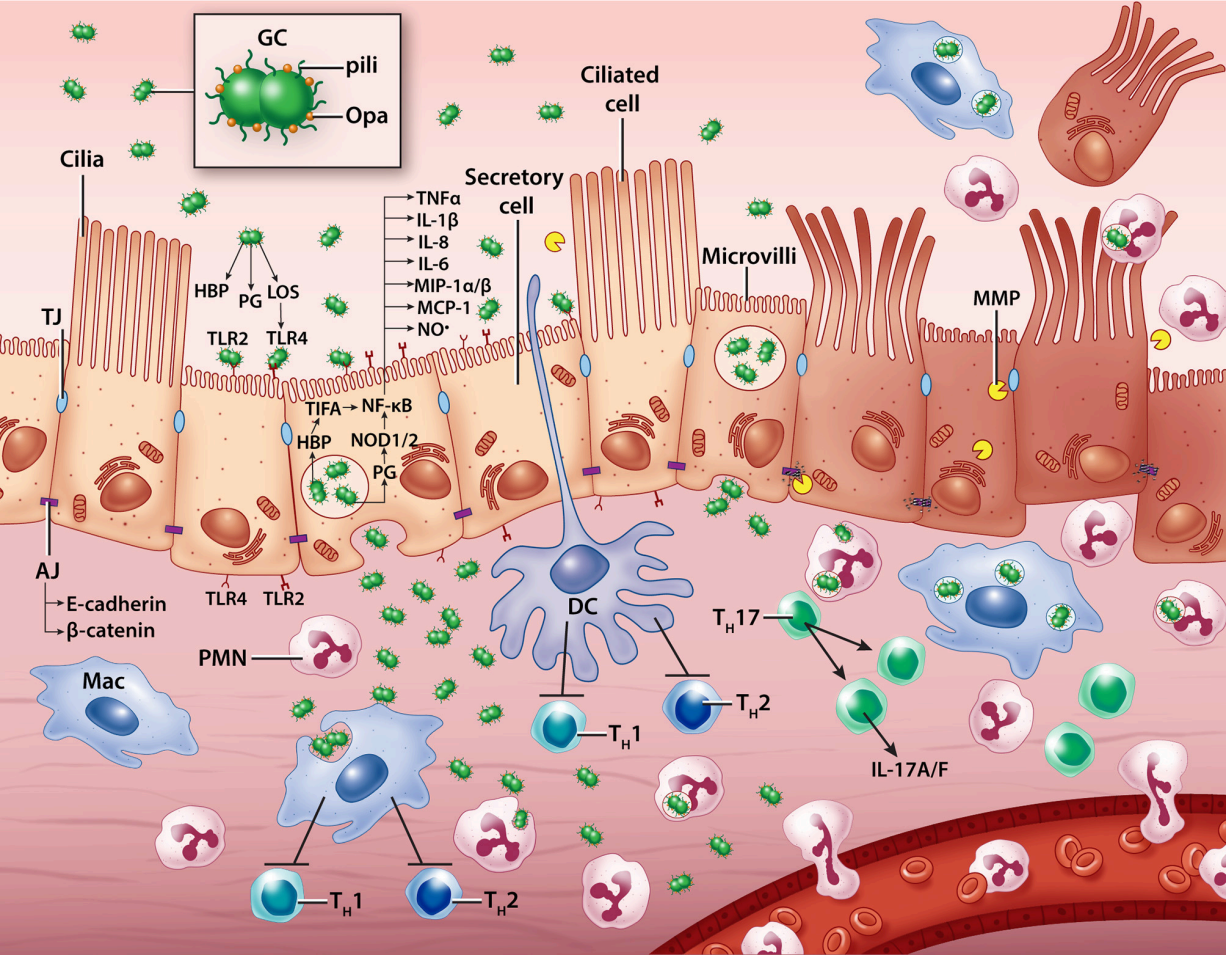
A model of *N. gonorrhoeae* pathogenesis in the human Fallopian tube. Extracellular gonococci interact with secretory (non-ciliated) epithelial cells, inducing cytokine, chemokine, nitric oxide, and matrix metalloproteinase production. Some bacteria transcytose through epithelial cells to invade the subepithelial space where they likely encounter resident macrophages, dendritic cells, and neutrophils which can help clear infection. Immune cells are also influenced by contact with GC and the building epithelial immune response to promote pro-inflammatory T cell activity and discourage productive memory T cell responses. Ciliated cells die and are sloughed from the epithelium, possibly with the help of GC-induced host processes that disassemble cell-cell junctions. GC, gonococcus; PMN, polymorphonuclear leukocyte (neutrophil); Mac, macrophage; DC, dendritic cell; T_H_, T helper cell; LOS, lipooligosaccharide; PG, peptidoglycan; HBP, heptose-1,7-bisphosphate; TLR, Toll-like receptor; MMP, matrix metalloproteinase; TJ, tight junction; AJ, adherens junction; NO•, nitric oxide.

In their long evolution with humans, gonococci have evolved methods to evade our most potent and useful defenses. GC can survive on and in epithelial cells, while facilitating passage through epithelial layers. GC is able to suppress the activity of dendritic cells and macrophages, and induce regulatory T cells to prevent CD4^+^ T cell responses. All of this suppression occurs while triggering immune responses through TLR2, TLR4, NOD1, NOD2, TIFA, and cGAS/STING to produce an inflammatory state. When neutrophils and macrophages arrive to clear gonococci, bacteria manage to survive within these professional phagocytes and, in some cases, promote cell death. Within this cytokine/chemokine milieu, pro-inflammatory Th17 cells are induced and Th17-dependent responses act to suppress Th1 and Th2 cells that would help generate immunologic memory. To date, we have assembled a working understanding of the complement of resident immune cells and distribution of receptors in the human fallopian tube. In addition, human fallopian tube explant experiments have revealed important properties of the innate immune response to GC, while animal models have contributed information on the adaptive immune response. By combining what is known from each of these different experimental sources, we are beginning to assemble a more complete picture of how GC promotes and exploits the inflammatory environment that causes PID (Figure 2).

## The “sterile” fallopian tube

The human female lower reproductive tract is an environment rich with commensal microbiota, containing around 10^8^ bacteria per gram of vaginal fluid (157), and dominated by Lactobacillus species in the majority of women (158). Once past the cervix, however, the uterus, fallopian tubes, and ovaries were thought to be effectively sterile to protect the reproductive process. Three recent studies have utilized quantitative polymerase chain reaction (qPCR) and/or Next-Generation Sequencing (NGS) of 16S ribosomal RNA (rRNA) genes to detect bacteria in fallopian tubes (159–161). Two of these studies confirmed their findings with culture of fallopian tube-resident bacteria (159, 161). Bacteria above the cervix are indeed found at much lower abundance than below the cervix, by about four orders of magnitude. The Fallopian tube appears to contain a diverse community including members of the Gram-negative proteobacteria (Acinetobacter, Pseudomonas, Comamonas), Gram-negative anaerobes (Bacteroides), and Gram-positives (Clostridium, Enterococci, Staphylococci, Vagococcus, Proprionibacterium). Rather than being dominated by any one genus, both studies point to a more diverse polymicrobial community trending toward aerobes and facultative anaerobes that prefer slightly alkaline conditions. As with the vaginal microbiome, the microbiome analyses of the Fallopian tube do not overlap perfectly in their findings, variations that may be accounted for by geographic or ethnographic differences (one study featured 110 reproductive-age women from Shenzhen, China, another just 16 women from Brisbane, Australia).

In the lower FRT, Lactobacilli are known to prevent gonococcal establishment and colonization through multiple mechanisms. Lactobacilli keep the vaginal pH low, produce inhibitory H_2_O_2_ (162), compete for binding sites on epithelial cells (163), and produce surface molecules that control GC in a contact-dependent manner (164, 165). Once infection is established at the cervix and has ascended as far as the fallopian tube, it is unclear if resident microbes have any effect on the progression of inflammatory disease. The identification of a resident microbial community, however, suggests an additional variable to consider, on top of host genetics, when considering an individual's risk of negative outcomes from gonorrhea.

## Thinking outside the tube

For human female upper reproductive tract infections, *ex vivo* or *in vitro* methods are the only ethical means for studying direct interactions between gonococci and their native host. Primary human-derived tissue is the nearest substitute for natural infection, but other alternatives either currently exist or could be applied to the fallopian tube given the state of the art for other tissues. Alternative technologies include human-derived primary cells, organoids (three-dimensional single or mixed cells), or organs-on-a-chip (OOC). Primary cells have been useful, to date, in the study of gonococcal infection in both males and females. Cells have been successfully derived from many of the principal tissues encountered during infection including cervical epithelium (166), endometrium (167), fallopian tube (60), and male urethral epithelium (168). Key findings regarding differences in receptor engagement and inflammatory responses between males and females (as mentioned above) have been determined using primary cells. The principle drawback to primary cells is the loss of multicellularity characteristic of intact tissues. In tissues, multiple stratified layers may be involved in immunological cross-talk or in engaging directly with bacteria during the progression of infection. In addition, adaptation to passage in cell culture and the loss of normal development/differentiation signals can impact the performance of primary cells in culture. For fallopian tubes in particular, the mixture of ciliated and non-ciliated cells, and the differences in their behavior during infection is key to the observed pathology.

Modern methods for isolating epithelial cells from Fallopian tube have not emerged from the field of infectious disease, but rather from studies on the origins of ovarian cancer, in particular high-grade serous ovarian carcinoma (HGSOC) (169). The desire to have models for studying oncogenic transformation of fallopian tube secretory cells has led to the development of isolation methods for primary fallopian tube secretory epithelial cells (FTSECs) and immortalized lines derived from these primary cells (170). Unfortunately, a side effect of fallopian tube epithelial cell isolation is a gradual loss of ciliated cells from culture upon passage (169). Indeed, ciliated cells appear to be a terminally differentiated cell type. In the murine airway epithelium, stem/progenitor cells known as basal cells give rise to secretory epithelial cells, which can then undergo terminal differentiation into ciliated cells (171). The maintenance of a secretory phenotype in this system is dependent on continuous signaling through the intercellular signaling receptor Notch2, without which secretory cells become ciliated cells. A similar linage relationship was also established for the epithelial cells of murine oviduct, where secretory cells are capable of self-renewal, as well as capable of differentiating into ciliated cells (172). In the oviduct, the balance of Wnt/β-catenin signaling is critical in both the maintenance of the secretory cell population and the differentiation into ciliated cells. While the human fallopian tube may not directly replicate the murine system, understanding the signals that are needed to maintain healthy mixed populations in culture allows us to improve how we establish and maintain *in vitro* models. Host developmental signaling pathways themselves may also be targets for pathogens, as evidenced by the ability of *C. trachomatis* to disrupt Wnt signaling in infected (and neighboring) epithelial cells in fallopian tube explants (100).

Defining an epithelial stem cell niche in the oviduct and determining the signals necessary to promote ciliated and secretory cell differentiation raises the possibility of using a patient's own stem cells to correct damage. Such repair could potentially restore fertility following tissue injury incurred from an STI or chemotherapy. Stem cells also provide additional possibilities for the generation of human-derived research materials. Induced pluripotent stem cell (iPSC) lines have been used to generate fallopian tube epithelium *de novo* (173). iPSCs first need to be induced to differentiate into intermediate mesoderm-like cells, and can subsequently be treated with Wnt pathway agonists (Wnt4 and Follistatin) to generate a fallopian tube epithelium that contains both ciliated and secretory cells. It is also possible to isolate multipotent mesenchymal stem cells (MSCs) from whole human fallopian tube tissue, as well as a subset of less proliferative MSCs from the mucosal portion of the tissue alone (174). These cells are capable of differentiation on adipogenic, osteogenic, and chondrogenic pathways and produce cytokines such as GM-CSF, IL-4, IL-6, TNFα and IFNγ. Kessler et al. demonstrated the isolation of adult stem cells from fallopian tube tissue, as well as defined the necessary culturing process to generate epithelia with a mixture of ciliated and secretory cells (175).

Successful culture of primary cells, stem cells, and/or iPSCs often requires suspension in (or grown on) an extracellular matrix (frequently Martigel, derived from Engelbreth-Holm-Swarm cells or minimally a collagen- or fibronectin-coated substrate). When placed in three-dimensional culture, rather than when adhered to a cell dish in monolayer, a single cell type (given proper inputs) can develop distinct polarity and cellular differentiation. These differentiated, three-dimensional cell clusters are known as spheroids or organoids. To generate faithful reproductions of fallopian tube epithelia, both iPSCs (173) and adult stem cells from dissected fallopian tube (175) need to be grown as organoids. Organoids hold tremendous promise for the study of complex cellular systems: they have the richness of signals provided by multiple cell types, spatial growth cues that monolayer cells lack, and they can be expanded in culture over long periods of time. Kessler *et al*. propagated continuously dividing, morphologically stable fallopian tube organoids for 10 months. In particular, for pathogens where animal models are lacking or do not faithfully recapitulate key aspects of infection, organoids can provide a middle ground between cell culture models and the natural infection seen in humans (176). Organoid systems have already gained traction for the study for intestinal pathogenic bacteria (177), where intestinal organoids (also known as enteroids) have been used to study bacterial adherence and invasion. For gonococci, where humans are the only natural host, the development of organoids that recapitulate the tissue architecture of fallopian tube present an exciting development and a promising future direction for host-pathogen interaction research. Since organoids can be derived from single (stem) cells, genome editing technologies including CRISPR/Cas9 could be used to alter host genes targeted during infection, prior to the growth of cells into mature organoids. These techniques have already been applied to edit the genomes of both adult stem cell- and pluripotent stem cell-derived organoids to study a variety of human diseases reviewed in (178, 179).

Another technology that could be used to replicate *in vitro* the biological complexity seen *in vivo* is organs-on-chips (OOCs), which come from a fusion of biological and engineering fields. OOCs are composed of either cell lines or primary cells from one or more tissues, cultured in custom-engineered microfluidic devices that can mimic multi-organ systems and incorporate complex biomechanical features like gas-exchange or liquid mixing (180). While OOC technology has room to improve in handling patient-derived or induced-pluripotent stem cells, microfluidic devices generally produce more reproducible results than organoids, which rely on meticulous culturing and development. Recently, a microfluidic model was constructed for the human female reproductive tract that was capable of mimicking the menstrual cycle (181). During ascending female reproductive tract infection that results in PID, gonococci must presumably interact with multiple cell types in sequence (endo/ectocervix, uterus, and fallopian tube) en route from their inoculation site. Having an interconnected, multicellular system would allow for the study of bacteria-host interactions throughout the process of gonococcal ascension to the fallopian tube. It is also unclear what bacterial or host factors predispose women to an asymptomatic cervical infection and whether this relates to their likelihood of progression to upper FRT infection. One possible contributing factor is hormonal cycles, which dramatically influence the tissue environment encountered by bacteria, and could be replicated in OOC systems. Hormonal changes are known to impact (1) primary human ectocervical epithelial cell (HECEC) responsiveness to peptidoglycan and LPS (182), (2) gonococcal survival during primary human cervical epithelial (pex) cell infection (51), and (3) the adherence and internalization rates for gonococci on fallopian tube epithelial cells (78). However, the full impact of hormonal signaling is difficult to assess in models that feature only one cell type or even one multicellular tissue. As technology evolves, more opportunities will exist to develop robust, flexible, *in vitro* models that retain the relevance of experimenting on primary human tissue.

## Concluding remarks

*Neisseria gonorrhoeae* remains a challenging organism for undertaking studies of host-pathogen interactions owing to its evolutionary adaptation to a life cycle in human hosts. However, the increasing utility of mouse models that incorporate human components, the availability of surgical samples from human tissues, and more adaptable human-like model systems, together provide great promise for understanding the biology of an important human pathogen. The use of fallopian tube explants provides a unique window into natural infection. Studying its multicellular epithelium has revealed how infection is linked to decreased fertility via bacteria- and host-factor toxicity to ciliated cells. Its three-dimensional structure has allowed observations about the speed of cellular invasion, the process of transcytosis and colonization of the sub-epithelial space. Thanks to insights from the fields of developmental biology, cancer biology, and gynecology, we know more than ever before about the process of ciliated cell maturation, how ciliated cells are maintained in tissues, and what immune responses are most likely to take place in the fallopian tube.

There are still several gaps that remain in our understanding of the molecular mechanisms that underlie gonococcal salpingitis. Chief among them are the cellular events surrounding ciliated cell death and extrusion from the epithelium. Prior to cell death, cellular changes result in a decrease in ciliary beat frequency (CBF). Loss of ciliated cell activity then precedes visible death and sloughing of ciliated cells. These phenomena have been difficult to measure, as the most widely accepted methods for quantitatively studying CBF involve high-speed video microscopy. Standard video microscopy is not ideal for whole tissue samples that lack optical transparency, or for monitoring changes in CBF across multiple time points in multiple samples. Many of the early works that describe decreases in CBF during gonococcal infection, and form the core of our knowledge on that topic, rely on essentially subjective visual estimates such as “percent peripheral ciliary activity (PPCA)” or “ciliary vigor” (15, 25). Future advances in live-cell, non-destructive, three-dimensional imaging of surfaces, such as light-sheet microscopy (183, 184), or methods that allow real-time *in vivo* or *ex vivo* measurements such as micro-scale tomography (185), will hopefully provide more robust measurement methods. With quantitative measurement methods, we can better probe what factors precipitate loss of CBF and what cellular mechanisms are involved in ciliated cell death and extrusion.

To understand how gonococcal infection results in permanently damaging salpingitis, we still need a clearer idea of how gonococci ascend into the fallopian tube. Current hypotheses include bacterial twitching motility, gonococcal survival inside phagocytes, or transport on sperm cells (186). We also need to understand the interplay of host factors that may predispose some women to more detrimental outcomes of ascending infection. Certainly, evidence has been presented that suggests differences in timing of infection during the menstrual cycle may influence the chance of negative outcomes. Additional investigation of host polymorphisms in immune receptors or adhesion ligands and their effects on gonococcal salpingitis represents an area for potential future investigation, especially with the increased accessibility of human genome sequencing. For example, some evidence exists that TLR2 polymorphisms and potentially other host differences can affect the outcome of *C. trachomatis* infection (187, 188). Understanding patient history, including more accurate diagnosis and documenting of instances of reproductive tract infection, is especially important now that we are aware of epigenetic mechanisms that influence future epithelial responses to repeated inflammatory stimuli (189, 190). At the center of any discussion of how to model infectious diseases should be a clear understanding of how the knowledge we gain into basic biological mechanisms translates into the features we see (or seek to prevent) in human disease. Fallopian tubes, and other human tissues, provide an excellent model system for answering certain questions about how gonococci interact with, and provoke immune responses from diverse human genetic backgrounds. Along with cellular and whole-animal systems, organ cultures represent a vital piece of the research puzzle that together allows us to assemble a more complete picture of *N. gonorrhoeae* pathogenesis.

## Author contributions

JL and JD conceived and outlined the manuscript. JL wrote the manuscript and designed figures. JD edited the manuscript and figures.

### Conflict of interest statement

The authors declare that the research was conducted in the absence of any commercial or financial relationships that could be construed as a potential conflict of interest.
